# T-SMmOTE: tweaked synthetic majority minority oversampling technique for data scarcity issue in multi omics studies

**DOI:** 10.1093/bioadv/vbag236

**Published:** 2026-08-14

**Authors:** Payel Sadhukhan, Animesh Acharjee

**Affiliations:** Department of Business Analytics, Army Institute of Management Kolkata, Newtown 700160, West Bengal, India; Department of Cancer and Genomic Sciences, University of Birmingham, Birmingham B15 2TT, United Kingdom

## Abstract

**Motivation:**

Multiomics data offer a rich data mine for modeling complex as well as day-to-day diseases, but their practical deployment is constrained by the limited sample availability. To this end, generating synthetic samples is a viable remedy. Extant schemes operating along this line, however, are mostly limited to augmenting the minority class in imbalanced datasets and often produce synthetic samples that lack sufficient diversity and fail to faithfully capture the underlying data distribution. As a result, the full potential of synthetic augmentation in multi-omics learning remains underexplored. The aim is to address the data scarcity problem in multi-omics domain. We propose a synthetic oversampling framework, which is dedicated to addressing overall data scarcity in multi-omics datasets and the lack of diversity in synthetic samples. Contrary to conventional methods that restrict augmentation to minority classes and rely on interpolation of two neighbors, our method generates diverse yet distribution-aligned synthetic samples by interpolating three neighbors and extends this augmentation paradigm to the majority class. The framework first balances the dataset by generating synthetic minority samples, and subsequently augments the balanced dataset by oversampling both majority and minority classes.

**Results:**

Empirical evaluation on multi-omics data obtained from three heterogeneous health scenarios—inflammatory bowel disease, multi-organ dysfunction syndrome, and colorectal cancer—substantiates the utility of the proposed scheme in improving the predictive performance. The models trained on T-SMmOTE–augmented data achieve higher Matthews correlation coefficient values, along with improved $F_{1}$ scores for both majority and minority classes. Notably, oversampling of the majority class improves the cognition of the minority class as well. We also explore the consistency of the class distributions between the original and augmented class-specific datasets. These findings confirm the capability of our scheme to learn from small, high-dimensional multi-omics datasets and highlight its potential for non-invasive disease detection.

**Availability and implementation:**

https://github.com/payelu/TSMm.

## 1 Introduction

The integration of multiple biological layers—including genomics, transcriptomics, proteomics, metabolomics, epigenomics, and clinical phenotypes—to characterize complex diseases is fundamentally challenged by the scarcity and heterogeneity of multi-omics datasets (Feldner-Busztin *et al.* 2023). Although clinical and biological data are typically gathered with precision through instrument-assisted human efforts, they remain fragmented, limited in scale (Tarazona *et al.* 2020, Feldner-Busztin *et al.* 2023), and heterogeneous (Knevel and Liao 2023, Maleki *et al.* 2022). The inadequate availability of datasets of admissible size often comes in the way of meaningful analysis, whether through clinical investigations or machine learning (ML)-based methodologies (Canzler *et al.* 2020). The scarcity of subjects consequently leads to small sample sizes, and when observations are recorded across a wide array of parameters, the complexity of analysis intensifies—often resulting in a high-dimensional, low-sample-size (HDLSS) scenario, commonly referred to as the p≫n problem (Lualdi and Fasano 2019, Shin and Oh 2024).

In such scenarios, ML techniques offer viable solutions, as their mathematical frameworks navigate this complexity and extract some meaningful patterns, even when data limitations constrict the formulation of fully optimal solutions (Mahmud *et al.* 2018, Montesinos-López *et al.* 2021). One prominent strategy along this line is synthetic data generation. Synthetic data generation is an artificial simulation of real-looking data through statistical methods (Chawla *et al.* 2002). In the biological domain, this class of methods is employed for a primary need: fairness of prediction. Class imbalance is a highly conspicuous issue in multiomics data; a natural bias arises from the differential prevalence of disease and symptoms (Han 2015). Models trained on such datasets are often suboptimal, as they develop bias against the quantitatively scarce minority class and prejudice in favor of the quantitatively abundant majority class. The underlying result is the failure to recognize data from the minority class, constituting a lack of fairness in the overall predictions (Yang and Mirzaei 2024, Han *et al.* 2025). To mitigate the issue, researchers generate synthetic minority points to obtain a balanced dataset and use it to build a fair model. An array of methods exists in the literature that explores diversified perspectives to generate synthetic *minority* points (Leevy *et al.* 2018). However, in multi-omics datasets, oversampling often distorts inter-omics correlations and intrinsic data distributions; naively applying synthetic sample generation to concatenated datasets may generate biologically implausible synthetic profiles (Salmi *et al.* 2024, Wang *et al.* 2025). A more fundamental challenge in multiclass datasets is the overall data scarcity, which gives rise to feasibility issues concerning the model. Learning a model from a small dataset imposes several limitations, the first and foremost being poor generalization and a randomized modus operandi.

In this work, we propose a scheme to address the aforementioned challenges by pursuing two objectives: (i) enhance diversity in synthetic samples while preserving the intrinsic distribution when generating the synthetic minority set, and (ii) alleviate the overall data scarcity by oversampling the majority class as well. To achieve the first goal, we adopt a three-point synthetic sample generation scheme. Unlike conventional approaches that rely on two neighbors, we use three nearest neighbors. By incorporating convex combinations of three neighboring points rather than two, the synthesized samples populate the interior regions spanned by the neighbors (instead of being restricted to points along the line segments connecting them). This geometric expansion augments both consensus and diversity, enhances coverage of the feature space, mitigates sampling bias, and exposes the learning algorithm to a richer array of feature variations. To cater to the latter, we extend this oversampling mechanism to the majority, thereby augmenting all the underrepresented classes in a small dataset. Our aim is to address the limited availability of samples in the multi-omics domain. We propose Tweaked Synthetic Majority minority Oversampling Technique (T-SMmOTE), whose modus operandi addresses the above-stated pitfalls and simultaneously augments both the majority and minority classes.

We conduct comprehensive experiments on datasets covering three different health scenarios: inflammatory bowel disease (IBD), multi-organ dysfunction syndrome (MODS), and colorectal cancer (CC). The choice of datasets is motivated by their distinct distribution profiles—small yet heterogeneous sample sizes, their diverse imbalance ratios, and the nature of the health conditions. The empirical evaluation using Matthews correlation coefficient (MCC) scores, majority-class $F_{1}$, and minority-class $F_{1}$ shows that the augmented datasets yield a significant overall improvement. The gains are substantial and consistent across both classes, highlighting the utility of the augmentation for learning complementary patterns. To assess the robustness of the proposed framework, we compute the distributional fidelity of the augmented data through a maximum mean discrepancy (MMD)-based analysis (Borgwardt *et al.* 2006). We examine the class-specific shifts between the original and augmented class distributions across the three datasets. The results, together, substantiate TSMmOTE’s utility in enabling non-invasive disease detection.

## 2 Methods

**Figure 1:**
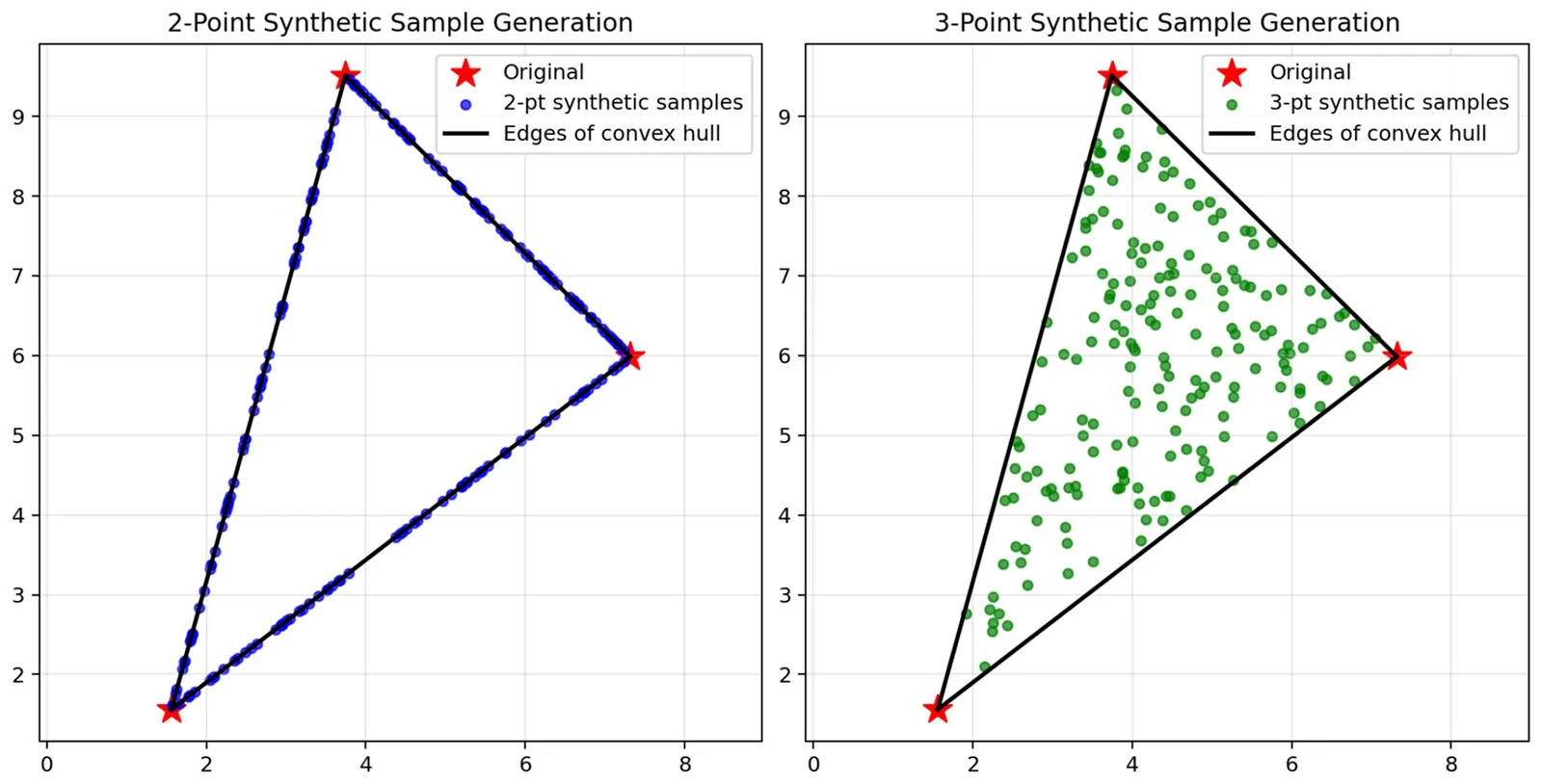
We have three original points in the given two-dimensional space, denoted by red stars. The goal is to obtain synthetic minority points (green points) from these three points. The left panel shows synthetic minority points generated through the convex combination of two original points; these points do not fill the entire feature space and are confined to the edges. The right panel shows synthetic minority points generated by the convex combination of all three points; these points fill the entire enclosed space, rendering a diverse and representative synthetic point set.

In this work, we address two fundamental challenges in multi-omics datasets: (i) limited sample sizes and (ii) the lack of diversity in synthetic samples. In the extant literature, methods have focused on improving the visibility of the underrepresented class rather than the entire dataset (Kaur *et al.* 2019). A significant number of the methods mitigate the issue by generating synthetic minority points as convex combinations of two original minority points (Leevy *et al.* 2018). The schemes, although effective at quantitatively mitigating class imbalance, confine synthetic points to a line joining the two points involved and significantly restrict the diversity of the synthetic data. Refer to Fig. 1.

To address this limitation, we employ a three-point data generation strategy; we generate the point as a convex combination of three original points. This extension shifts the generative region from a two-dimensional line to a three-dimensional planar surface—thereby enhancing the diversity of the synthetic samples and coverage of the feature space.

Building on this foundation, we propose a method, TSMmOTE. Our method pursues two distinct objectives—(i) augmenting the diversity of the synthetic instances without perturbing their distribution, and, (ii) enhancing the representation of both minority and the majority classes of a dataset to mitigate the overall data scarcity.

TSMmOTE operates in two distinct stages. The first stage of TSMmOTE employs the three-point method to generate a diverse set of synthetic minority points, effectively balancing class distributions while preserving and expanding upon the topological features of the original minority data. The second stage addresses overall data scarcity by performing distribution-aware synthetic set generation (and selection) for both classes, thereby magnifying representation while maintaining statistical and structural integrity.

Let D=(X,Y) be the original dataset. It consists of a feature vector $X\in R^{n\times p}$, where usually n≪p, and the class label vector $Y\in R^{n\times 1}$. $x_{i}\in X$ represents the $i^{\text{th}}$ point of the dataset D, and $y_{i}$ is its corresponding class label. We consider binary datasets in our work, hence, $y_{i}\in \{0,1\}$. The minority set (or class), $X_{m}$ consists of all points $x_{i}\in X$ for which $y_{i}=1$. Similarly, the majority set, $X_{M}$ consists of all points $x_{i}\in X$ for which $y_{i}=0$. Since D is imbalanced: $\left|X_{m}|\ll |X_{M}\right|$, and together they constitute the entire dataset: $|X_{m}|+|X_{M}|=n$.


$$X_{m}=\{x_{i}\in X|y_{i}=1\}$$

$$X_{M}=\{x_{i}\in X|y_{i}=0\}$$

### 2.1 Balancing the classes

To balance the classes, we employ a tweaked version of SMOTE that employs three neighbors instead of two neighbors. The procedure yields a synthetic minority point set, $S_{m}$. The balanced dataset is formed by taking the union of the majority set $X_{M}$, the minority set $X_{m}$, and the synthetic minority set $S_{m}$.

$X_{m}=\{x_{1},x_{2},\ldots ,x_{N_{m}}\}$ is the minority set. A synthetic point $\tilde{x}$ is generated as:


$$\tilde{x}=\sum_{i=1}^{k}w_{i}x_{i},\;\text{where}\;x_{i}\in X_{m},\;w_{i}\geq 0,\;\sum_{i=1}^{k}w_{i}=1$$

$x_{i}\in X_{m}$ implies that the original minority points are used to generate the synthetic minority point. $w_{i}\geq 0$ determines the contribution of each minority point in generating the synthetic minority point; the higher the weight, the higher its similarity with the synthetic point.

In this work, instead of involving two minority points in the generation of the synthetic minority point, we set k=3 and use three points:


$$\tilde{x}=\lambda x+\mu r_{1}+\nu r_{2}$$

where λ,μ,ν are non-negative weights satisfying the convex combination constraint:


λ+μ+ν=1, λ,μ,ν≥0

The cardinality of $S_{m}$ is equal to $\left(|X_{M}|-|X_{m}|\right)$. The balanced dataset B is obtained by taking the union of $X_{M}$, $X_{m}$, and $S_{m}$:


$$B=X_{M}\cup X_{m}\cup S_{m}$$

B has equal sizes of the majority and the minority classes. Despite that, both classes are underrepresented.

#### 2.1.1 *Dimensionality reduction via SVD*

The datasets pertaining to our study have very high dimensionality in the range of thousands and are prone to the shortcomings related to high dimensionality. To address this, we apply truncated singular value decomposition (SVD) independently on the two classes, as their characteristics are distinct from one another. Let $X_{C}\in R^{n_{C}\times p}$ denote the feature matrix of class C∈{m,M}, where $p\gg n_{C}$ in the HDLSS setting. Under the thin decomposition, SVD factorizes $X_{C}$ as:


$$X_{C}=U_{C}\Sigma_{C}V_{C}^{⊤}$$

where $U_{C}\in R^{n_{C}\times n_{C}}$, $\Sigma_{C}\in R^{n_{C}\times n_{C}}$ is the diagonal matrix of singular values $\sigma_{1}\geq \sigma_{2}\geq \ldots \geq \sigma_{n_{C}}\geq 0$, and $V_{C}^{⊤}\in R^{n_{C}\times p}$. We retain the top-$d_{C}$ components that collectively explain at least 90% of the total variance in class *C*:


$$d_{C}=\text{min}\left\{d:\frac{\sum_{i=1}^{d}\sigma_{i}^{2}}{\sum_{i=1}^{n_{C}}\sigma_{i}^{2}}\geq 0.90\right\}$$

The reduced representation is obtained by projecting $X_{C}$ onto the top-$d_{C}$ right singular vectors:


$${\tilde{X}}_{C}=X_{C}V_{C}^{\left(d_{C}\right)}\in R^{n_{C}\times d_{C}}$$

where $V_{C}^{\left(d_{C}\right)}\in R^{p\times d_{C}}$ comprises the top $d_{C}$ right singular vectors of $X_{C}$. The SVD basis is fitted exclusively to the original class samples and not to the synthetic pool, ensuring that the reduced space reflects the intrinsic variance structure of the original data rather than any distributional characteristics introduced by the synthetic generation process. This renders the reduced representation invariant to differences in sample size between the original and synthetic pools. This dimensionality reduction serves two purposes in the TSMmOTE framework. First, it mitigates the instability of distance-based computations in high-dimensional spaces where $p\gg n_{C}$, as nearest-neighbor relationships and Wasserstein distances become unreliable when the number of features substantially exceeds the number of samples. By projecting into a compact, variance-preserving subspace, we ensure that the subsequent RkNN analysis and Wasserstein-based elite subset selection operate on geometrically meaningful distances. Second, the 90% variance retention threshold is chosen to balance noise suppression and information retention.

Note that this dimensionality reduction is applied solely for the purposes of RkNN analysis and Wasserstein-based elite subset selection. The synthetic points themselves are generated and retained in the original *p*-dimensional feature space, ensuring that the final augmented dataset R possesses the full feature dimensionality required for subsequent model training and prediction.

### 2.2 Augmenting the balanced dataset

The key focus of this work is to address the limited sample size problem in datasets. B, although balanced in terms of class representation, exhibits overall under-representation of the constituent classes. Each class is represented by a limited number of samples, which affects the cognitive power and reliability of the model trained on it.

To mitigate this, we propose to generate synthetic points for all classes of the dataset. The method proceeds through four phases for each class: candidate pool generation, subset sampling, elite subset selection via Wasserstein distance, and consensus-based point selection.

The procedure generates two sets: (i) a synthetic majority point set, $R_{M}$, and (ii) a synthetic minority point set, $R_{m}$, of the required cardinality. In this work, the cardinalities are kept equal for both classes, with $|R_{M}|=|R_{m}|=\frac{\delta \times |B|}{2}$, where δ is the degree of augmentation. The pool serves as the initial set of synthetic samples, from which we select the most suitable points for model training. The objective of the refinement stage is to select those synthetic points that show a high degree of homogeneity with their respective distributions. By filtering out poorly aligned synthetic points, the refinement process ensures that only representative, reliable samples contribute to the final augmented dataset, thereby improving the robustness and discriminative power of the classifier.

#### 2.2.1 Generating a pool of synthetic instances

We use a three-way SMOTE (as in the previous subsection) to generate the synthetic instances. While generating a synthetic minority or majority point, we select a point from its respective class, say, $x^{*}$. We then randomly choose two of its nearest neighbors from the same class, $r_{1}$ and $r_{2}$. The synthetic point s is computed as a convex combination of $x^{*}$, $r_{1}$, and $r_{2}$:


$$s=\lambda x^{*}+\mu r_{1}+\nu r_{2}$$

where λ,μ,ν are non-negative weights satisfying the convex combination constraint:


λ+μ+ν=1, λ,μ,ν≥0

Let the pool of synthetic majority and minority instances be represented by $P_{M}$ and $P_{m}$, respectively. We will select an augmented and refined set of synthetic majority and minority points $R_{M}$ and $R_{m}$, respectively, from $P_{M}$ and $P_{m}$. Since we are focused on the balanced representation of the two classes, we keep $|R_{M}|=|R_{m}|=\frac{\delta \times |B|}{2}$ and $\left|P_{M}|=|P_{m}|=\theta \times |B\right|$, where θ>δ ensures that the pool is sufficiently large for meaningful subset selection. The utility of the three-point synthetic sample generation over two-point synthetic sample generation (as in state-of-the-art oversampling techniques) is illustrated in Fig. 1. The rest of the procedure is carried out in a class-specific manner.

### 2.3 Subset sampling for both classes

From the candidate pool of each class, we systematically sample multiple subsets to explore the potential of the synthetic samples. We apply the sampling procedure independently to both minority and majority classes:


$$SS_{t}^{m}\subset P_{m},\;|SS_{t}^{m}|=l,\;\text{with}\;N_{m}\leq l\ll |P_{m}|$$

for t=1,…,T, where *T* is the total number of subsets sampled per class, and $N_{m}$ is the number of original minority instances. Similarly, for the majority class:


$$SS_{t}^{M}\subset P_{M},\;|SS_{t}^{M}|=l,\;\text{with}\;N_{M}\leq l\ll |P_{M}|$$

The sampling scheme generates a diverse set of candidate subsets for each class; the goal is to evaluate the alignment of the synthetic points with their respective original class distributions.

#### 2.3.1 Class-specific elite subset selection through Wasserstein distance

To estimate the class alignment of the subsets and the points in them, we compute the reverse nearest neighborhood of the points. We perform RkNN analysis and elite subset selection independently for each class. For the minority class, we compute the RkNN count for each point in the original minority dataset ${\tilde{X}}_{m}$ (the SVD-reduced representation):


(1) $${\text{RkNN}}_{m}(x_{i}^{m})=\left|\left\{x_{j}^{m}\in {\tilde{X}}_{m}\setminus \{x_{i}^{m}\}:x_{i}^{m}\in {\text{kNN}}_{m}(x_{j}^{m},k)\right\}\right|$$

where ${\text{kNN}}_{m}(x_{j}^{m},k)$ denotes the set of *k* nearest neighbors of $x_{j}^{m}$ within ${\tilde{X}}_{m}$. We then select the top-*r* minority points to form:


(2) $$X_{\text{RkNN}}^{m}=\{x_{\left(1\right)}^{m},x_{\left(2\right)}^{m},\ldots ,x_{\left(r\right)}^{m}\}\subset {\tilde{X}}_{m}$$

Analogously, for the majority class, we compute:


(3) $${\text{RkNN}}_{M}(x_{i}^{M})=\left|\left\{x_{j}^{M}\in {\tilde{X}}_{M}\setminus \{x_{i}^{M}\}:x_{i}^{M}\in {\text{kNN}}_{M}(x_{j}^{M},k)\right\}\right|$$

and select:


(4) $$X_{\text{RkNN}}^{M}=\{x_{\left(1\right)}^{M},x_{\left(2\right)}^{M},\ldots ,x_{\left(r\right)}^{M}\}\subset {\tilde{X}}_{M}$$

The RkNN subsets serve as the reference lattices for the two classes. For each class C∈{m,M}, we compute the Wasserstein distance between its RkNN subset and each candidate subset from the corresponding synthetic pool:


(5) $$W_{p}(P_{X_{\text{RkNN}}^{C}},P_{SS_{t}^{C}})={\left(\underset{\gamma \in \Gamma (P_{X_{\text{RkNN}}^{C}},P_{SS_{t}^{C}})}{\text{inf}}\sum_{i=1}^{r}\sum_{j=1}^{l}\gamma_{ij}\|x_{i}^{C}-s_{j}^{C}{\|}^{p}\right)}^{1/p}$$

We then identify the elite subsets for each class independently. For the minority class:


$$E_{m}=\{SS_{\left(1\right)}^{m},SS_{\left(2\right)}^{m},\ldots ,SS_{\left(k\right)}^{m}\}$$

with ordered distances:


(6) $$\begin{array}{c} W_{p}(P_{X_{\text{RkNN}}^{m}},P_{SS_{\left(1\right)}^{m}})\leq W_{p}(P_{X_{\text{RkNN}}^{m}},P_{SS_{\left(2\right)}^{m}})\\ \leq \ldots \leq W_{p}(P_{X_{\text{RkNN}}^{m}},P_{SS_{\left(k\right)}^{m}}) \end{array}$$

For the majority class:


$$E_{M}=\{SS_{\left(1\right)}^{M},SS_{\left(2\right)}^{M},\ldots ,SS_{\left(k\right)}^{M}\}$$

with:


(7) $$\begin{array}{c} W_{p}\left(P_{X_{\text{RkNN}}^{M}},P_{SS_{\left(1\right)}^{M}}\right)\leq W_{p}\left(P_{X_{\text{RkNN}}^{M}},P_{SS_{\left(2\right)}^{M}}\right)\\ \leq \ldots \leq W_{p}\left(P_{X_{\text{RkNN}}^{M}},P_{SS_{\left(k\right)}^{M}}\right) \end{array}$$

**Figure 2:**
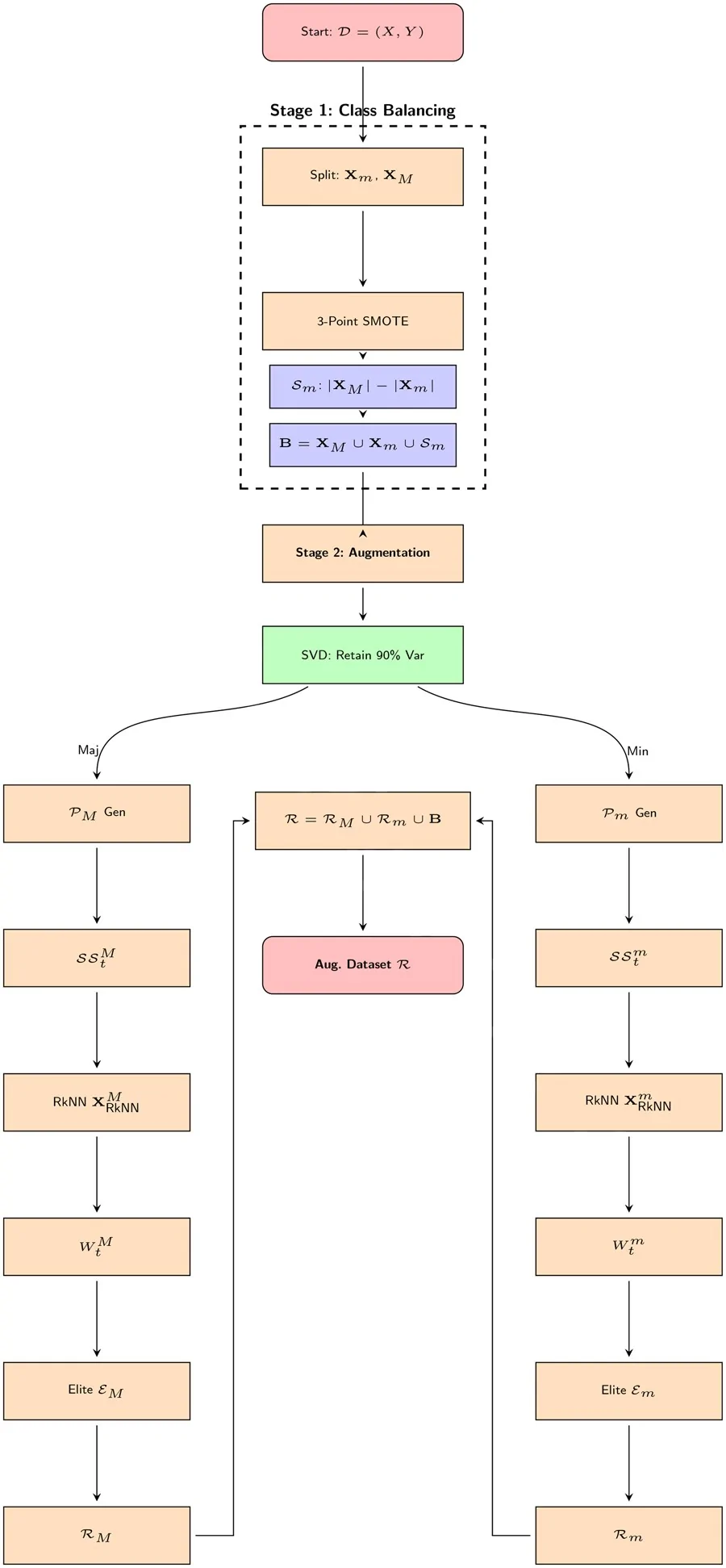
Workflow of TSMmOTE. Stage 1, balances classes using three-point oversampling. Stage 2, applies SVD to both classes to retain 90% of variance, then performs augmentation on the reduced feature space. It implements the novel protocol of majority oversampling, as well as the usual minority oversampling.

This class-specific elite subset selection is incorporated to ensure that the vitality and goodness of the synthetic points are based on their alignment with the structurally important regions of their respective original class distributions (Fig. 2).

#### 2.3.2 Class-specific consensus-based point selection

We perform consensus-based selection independently for each class. For the minority class, for each candidate point $c\in P_{m}$, we compute:


(8) $$f_{m}(c)=\sum_{j=1}^{k}w_{j}\cdot I(c\in SS_{\left(j\right)}^{m})$$

where $w_{j}=\frac{1}{j^{\rho }}$, with ρ>0 controlling the rate of decay of the subset rank weights. The top-$n_{m}$ minority points with the highest $f_{m}(c)$ scores form the refined minority representative set:


(9) $$R_{m}=\underset{R^{\prime }\subset P_{m},\;|R^{\prime }|=n_{m}}{\text{argmax}}\sum_{r\in R^{\prime }}f_{m}(r)$$

Similarly, for the majority class, for each $c\in P_{M}$:


(10) $$f_{M}(c)=\sum_{j=1}^{k}w_{j}\cdot I(c\in SS_{\left(j\right)}^{M})$$

and we select:


(11) $$R_{M}=\underset{R^{\prime }\subset P_{M},\;|R^{\prime }|=n_{M}}{\text{argmax}}\sum_{r\in R^{\prime }}f_{M}(r)$$

The sizes $n_{m}$ and $n_{M}$ can be determined from the desired class balance in the final dataset. Popular ways to obtain that include:

- Balanced distribution: $n_{m}=n_{M}$ for equal class representation
- Proportional distribution: $n_{m}:n_{M}=N_{m}:N_{M}$ to preserve original class ratios
- Minority-augmented distribution: $n_{m}>n_{M}$ to specifically address minority class under-representation.

In this research, we want a balanced dataset, hence we set $n_{m}=n_{M}$.

#### 2.3.3 Obtaining augmented dataset

We combine $R_{m}$, $R_{M}$, and the balanced dataset B to form the augmented dataset. It serves as the working data for building the classifier model.


(12) $$R=R_{m}\cup R_{M}\cup B$$

The statistical foundation of TSMmOTE is provided in the Appendix 1.

### 2.4 Datasets used in this study

In this study, we analyze three multi-omics datasets originating from three different health conditions: (i) IBD, (ii) MODS, and (iii) CC.

The first one is a multi-omics dataset from cross-continental cases of IBD (Franzosa *et al.* 2019). The cohort comprised 226 adult patients: 161 from Massachusetts General Hospital (USA) and 65 from the Lifelines DEEP study and University Medical Centre Groningen (The Netherlands). For each patient, fecal samples were profiled, yielding an initial feature set of 11 720 microbial genera (metagenomics) and 8848 metabolites (metabolomics). Patients were categorized by IBD diagnosis. The majority class consisted of 164 IBD cases (88 with Crohn’s Disease and 76 with Ulcerative Colitis), diagnosed via standard endoscopic criteria or prior clinical history. The minority control class contained the remaining 56 individuals without IBD.

The second transcriptomics dataset is publicly available and is based on a collection of prospective, observational Brain Biomarkers After Trauma Cohort Study (NRES 13/WA/0399), conducted at the Queen Elizabeth Hospital, Birmingham, UK (Hazeldine *et al.* 2017). The cohort consisted of 89 adult trauma patients with severe injuries, with Injury Severity Score (ISS) ≥ 8. The primary outcome was the development of MODS in these patients. It is quantitatively defined as a sequential organ failure assessment (SOFA) score ≥ 6 on 2 or more consecutive days, commencing at least 48 h post-admission. Peripheral blood samples were collected at three time points: within 1 h of injury (mean time: 42 min), 4–12 h post-injury, and 48–72 h post-injury. The probability of survival at 14 days (PS14) was estimated by trained staff from the Trauma and Audit Research Network. The PS14 model encompassed age, gender, ISS, the Charlson Comorbidity Index (CCI), 30-day patient outcome, and the Glasgow Coma Score (GCS); where GCS was missing, the variable for intubation was substituted. Detailed protocols for patient selection, informed consent, and blood sample parameters are publicly available. The class label is related to the development of MODS; patients were assigned a label of 1 if they developed MODS and 0 if they did not. There were 26 MOD samples and 35 non-MOD samples.

The third dataset is based on the detection of CC in patients through omics markers obtained from fecal samples (Bosch *et al.* 2022). Despite being fatal, early detection of the disease through screening is known to reduce mortality. The dataset used in this study is a small sample size one with 13 patients diagnosed with CC and 20 control samples; data were collected between February 2016 and November 2019. Information was collected via proteome, microbiota, and amino acid composition as an assessment of fecal samples prior to bowel movement. In addition to the bio-markers, demographic and lifestyle variables like age, gender, and smoking status of the participants were also recorded. These two data streams were integrated to create a comprehensive feature set for each sample, the aim is to improve the correctness of the non-invasive colorectal cancer detection.

The datasets used in this study present distinct class distribution profiles. In the first dataset, the diseased class (IBD) outnumbered the non-diseased control class, yielding an imbalance ratio of 2.71 (Table 1). The second dataset has a nearly balanced condition as the diseased class (MOD) only thinly outnumbered by the healthy class (non-MOD) rendering an imbalance ratio close to 1 (1.19). In contrast, the third dataset is characterized by the control class outnumbering the diseased class, rendering an imbalance ratio of 1.5. It also presents a unique, challenging scenario as its overall sample size is only 33.

**Table 1:** Summary of datasets, class distributions, feature composition, and data .

Dataset	Class distribution	Features	References
Inflammatory bowel disease (IBD)	IBD: 164 CD: 76, UC: 76 Control: 56	Metabolomics: 8848 microbiome: 11 720	Franzosa *et al.* (2019)
Multi-organ dysfunction syndrome (MODS)	MODS: 26 Non-MODS: 35 (ISS ≥ 8)	Transcriptomics (blood, 3 timepoints) Clinical: age, ISS, GCS, PS14	Hazeldine *et al.* (2017)
Colorectal cancer (CC)	CC: 13 Control: 20	Multi-omics: 225 OTUs, 521 proteins, 44 AA Demographics and biomarkers	Bosch *et al.* (2022)

AA: amino acid; OTU: operational taxonomic unit; ISS: Injury Severity Score; GCS: Glasgow Coma Scale; PS14: Probability of Survival.

A unique challenge connects the datasets; in each of them, the features/samples ratio >1. This scenario makes the models susceptible to improper learning through overfitting and data memorization. In this scenario, synthetic samples are required to improve the cognition of the classes and the learning of the classifier. The work focuses on generating meaningful synthetic instances for the overall dataset, not just the minority class, and calls for a synthetic sample generation scheme for all classes. The research objective of the study aligns with the challenges associated in these datasets, hence we include them in our exploration.

### 2.5 Evaluation methods

This section presents a comprehensive evaluation of the proposed TSMmOTE’s efficacy for learning from the described multi-omics datasets. The core experimental components, including (i) methods and their parameter configuration, (ii) metrics used for performance evaluation, and (iii) experiments and objectives, are presented in this section.

The performance of the proposed method, TSMmOTE, is benchmarked against seven other competing methods. The methods come from a diverse array of techniques: (i) state-of-the-art oversampling techniques: SMOTE (Chawla *et al.* 2002) and ADASYN (He *et al.* 2008), (ii) two relatively new oversampling techniques: Geometric SMOTE (Geom-SMOTE) (Douzas and Bacao 2019) and Generative Adversarial Network-based oversampling (GAN) (Scott and Plested 2019, Cusworth *et al.* 2024), (iii) synthetic sample refinement techniques: T-Tomek (Devi *et al.* 2017), T-ENN (Batista *et al.* 2004), and MAB-based refinement (MAB refinement) (Sadhukhan 2025). For SMOTE, ADASYN, and Geometric-SMOTE, we used their Python implementations in their default configurations. GAN consists of 64–32 neuron generator/discriminator networks, trained for 5000 epochs to synthesize minority samples for class balancing. For T-Tomek and T-ENN, we have undersampled the synthetic minority instances; for T-ENN, the neighborhood size is chosen as 3. MAB refinement is carried out at k=5, γ=20, and α=2.

We investigate all datasets and methods using five-fold cross-validation. To ensure a fair and consistent comparison, we use the same data partitions as TSMmOTE and all competing approaches. For each fold, we train models on the corresponding training subset and then use them to predict the held-out test points. This procedure is repeated across 50 independent runs for every dataset–method combination to improve statistical reliability. The aggregate results are reported in Section 5.

#### 2.5.1 Evaluating metrics

In this work, we aim to improve the overall learning from the multiomics dataset. To do the needful, we evaluate the performance on the basis of not only the minority class $F_{1}$, but also the majority class $F_{1}$. Additionally, we have reported the MCC scores obtained by the methods.

#### 2.5.2 Experiments and objectives

The experimental study is designed to evaluate the intrinsic efficacy of TSMmOTE for learning from multi-omics datasets. It has three key components.

- Comparative performance analysis: The primary experiment of this research benchmarks the performance of TSMmOTE against that of seven other competing methods. We may note that unlike the extant methods, the utility of the synthetic minority instances generated by TSMmOTE extends to the majority class as well.
- Analysis on preservation of distribution: Synthetic data generation forms the backbone of TSMmOTE. To understand the overall utility of this method, it is necessary to assess the distributional shift between the augmented distribution with respect to its corresponding original distribution. The second experiment of this research is dedicated to this aspect. We quantify this shift using MMD, a kernel-based two-sample statistic that measures the distance between the original and augmented class distributions, computed independently for the majority and minority classes. This empirical analysis complements the theoretical distributional convergence guarantees discussed in Appendix 1.
- Ablation study: The ablation study of this research is conducted in two dimensions. This research addresses learning from small datasets, for which augmentation is proposed as a remedy. We therefore conducted a dedicated ablation study to assess the degree of augmentation required by each dataset to achieve optimal performance. Specifically, we vary the degree of augmentation, the multiplicative factor by which the balanced dataset is expanded, from 2× to 10× the original cardinality, and identify the optimal value for each of the three datasets under study. Note that this study was conducted using the AdaBoost classifier. The second ablation study investigates the utility of TSMmOtE by comparing the performances of the learned models across three configurations: (i) models trained on the original dataset, (ii) models trained on the intermediate output of TSMmOTE (in which only synthetic minority instances are added), and (iii) models trained on the final output of TSMmOTE (in which both synthetic majority points and synthetic minority points are added). This analysis is conducted to isolate and quantify the value added by each distinct stage of the TSMmOTE framework. The goal is to elucidate how successive stages contribute to performance gains and enhance the overall effectiveness of TSMmoTE.

## 3 Results 

### 3.1 Experiment on comparative performance analysis

**Figure 3:**
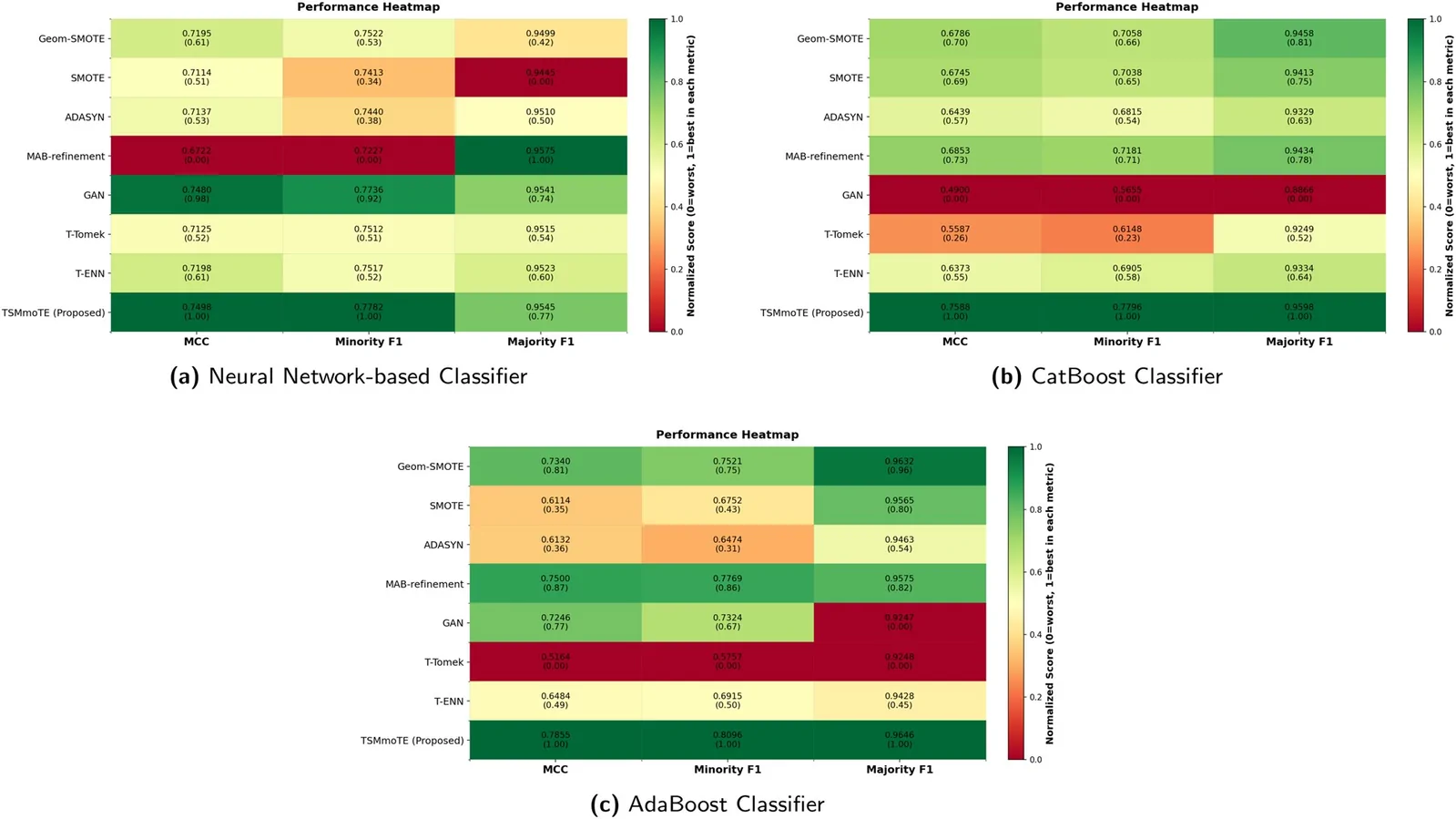
The figure shows comparative performance of the proposed method and seven competing methods on *IBD* dataset. (a)–(c) are dedicated to three classifiers. Each shows the performance of the methods on three metrics: MCC scores, minority-class $F_{1}$, and majority-class $F_{1}$. Both the raw scores and the normalized scores are shown, the colors are indicated according to the normalized scores. TSMmOTE has consistently outperformed the remaining methods across all but one scenario.

**Figure 4:**
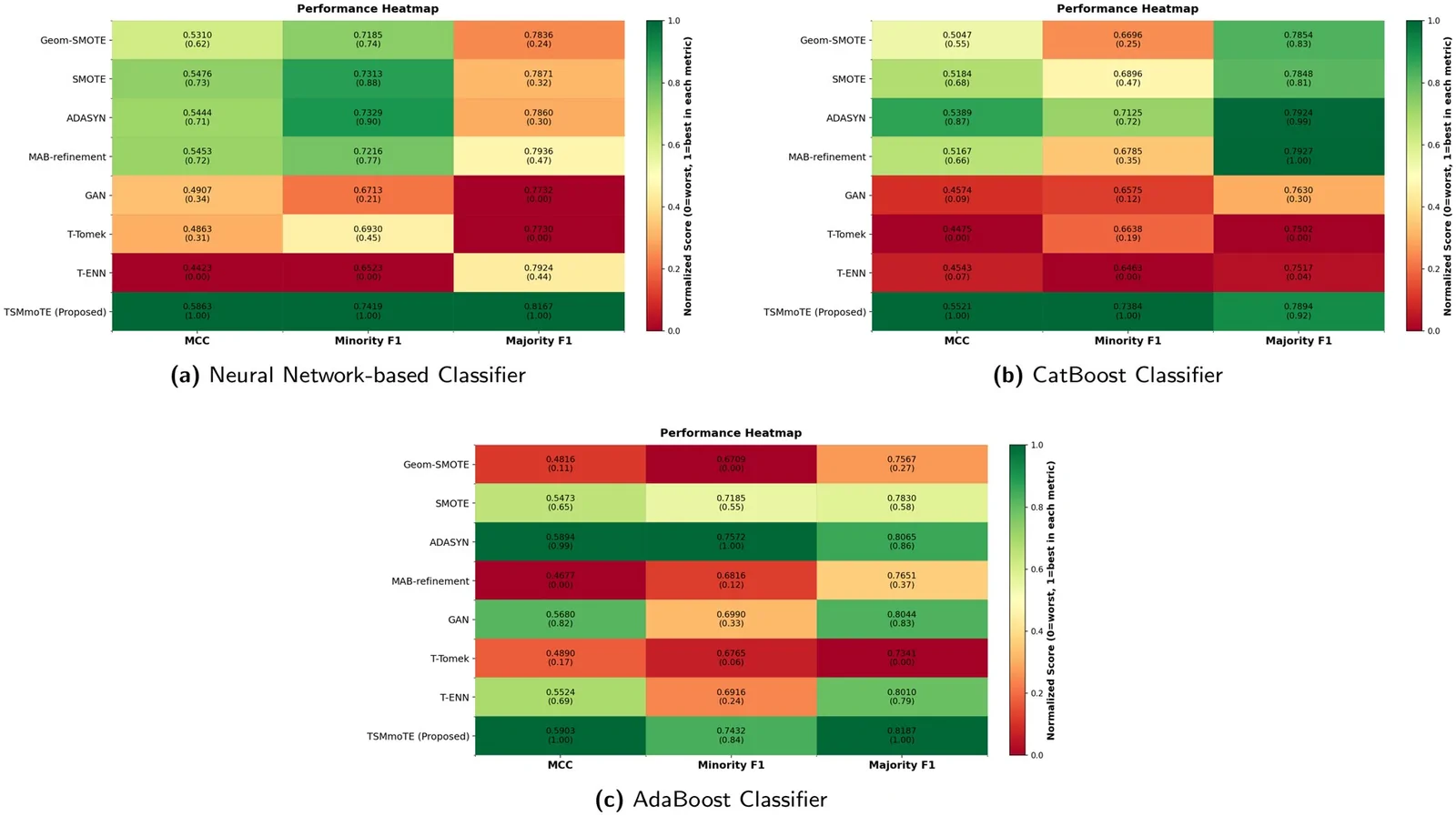
The figure shows comparative performance of the proposed method and seven competing methods on *MODS* dataset. (a)–(c) are dedicated to three classifiers. Each shows the performance of the methods on three metrics: MCC scores, minority-class $F_{1}$, and majority-class $F_{1}$. Both the raw scores and the normalized scores are shown, the colors are indicated according to the normalized scores. TSMmOTE has consistently outperformed the remaining methods across all but two scenarios.

**Figure 5:**
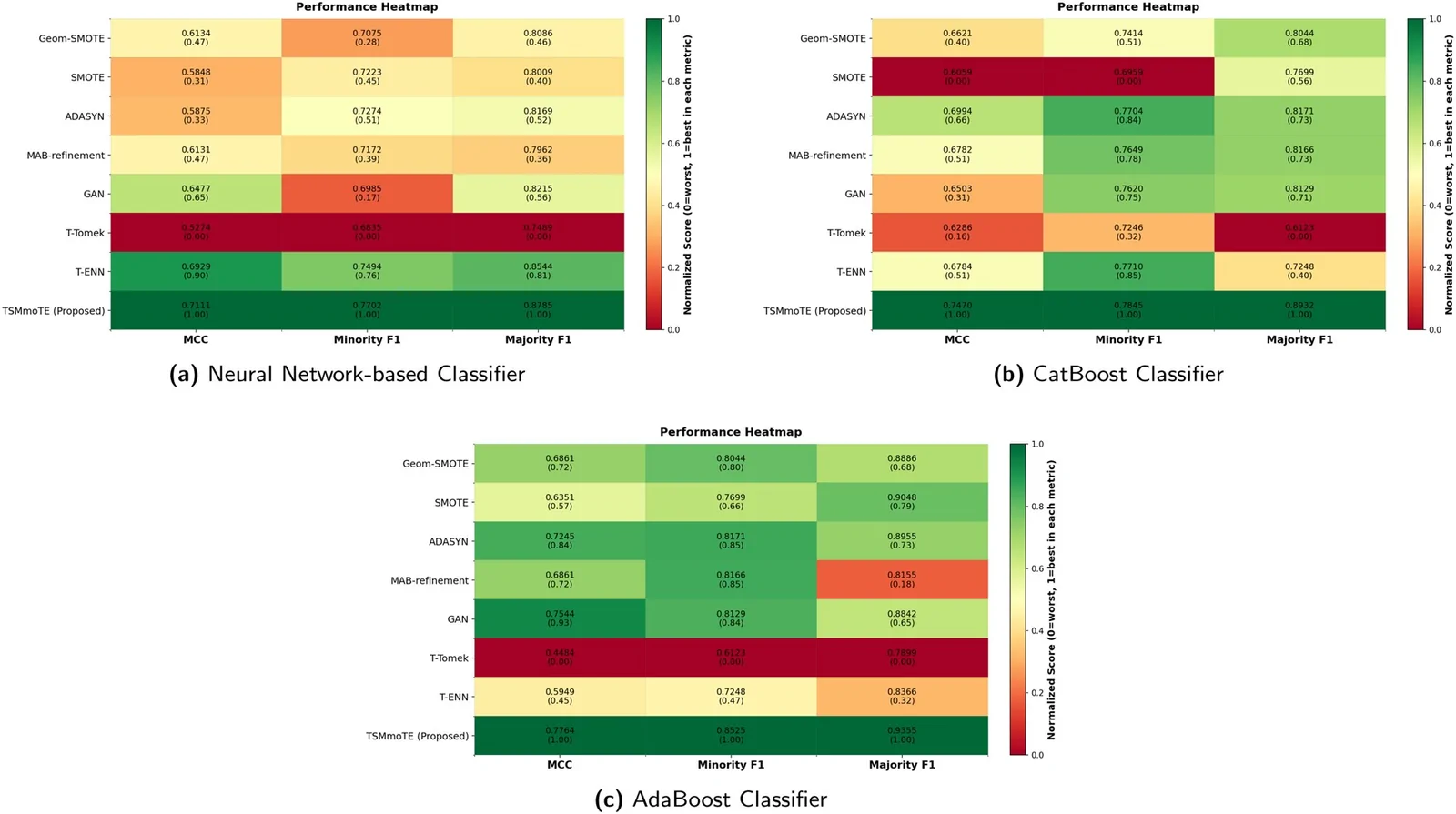
The figure shows comparative performance of the proposed method and seven competing methods on *COLORECTAL CANCER* dataset. (a)–(c) are dedicated to three classifiers. Each shows the performance of the methods on three metrics: MCC scores, minority-class $F_{1}$, and majority-class $F_{1}$. Both the raw scores and the normalized scores are shown, the colors are indicated according to the normalized scores. TSMmOTE has consistently outperformed the remaining methods across all scenarios.

In the primary experiment, we compare the performance of models trained on balanced or refined datasets obtained via various data preprocessing methods, as well as TSMmOTE. Figures 3–5 present the consolidated performance of the eight methods across three metrics and three diverse classifiers, with Figs. 3–5 dedicated to the IBD dataset, the MODS dataset, and, the CC dataset, respectively. On IBD dataset, TSMmOTE has demonstrated substantial superiority, achieving the best scores on eight out of the nine combinations of metrics and classifiers (three metrics × three classifiers). A similar, but slightly subdued, dominance is exhibited by TSMmOTE on the MODS dataset. TSMmOTE achieves all three best scores on Neural Networks Classifier but fails to obtain one each for CatBoost Classifier and AdaBoost Classifier. On the Colorectal Cancer dataset, TSMmOTE achieves all nine best scores. Overall, TSMmOTE secures 24 of the 27 total metric-classifier combinations across the three datasets, with the few exceptions occurring by narrow margins.

### 3.2 Analysis on preservation of distribution

**Figure 6:**
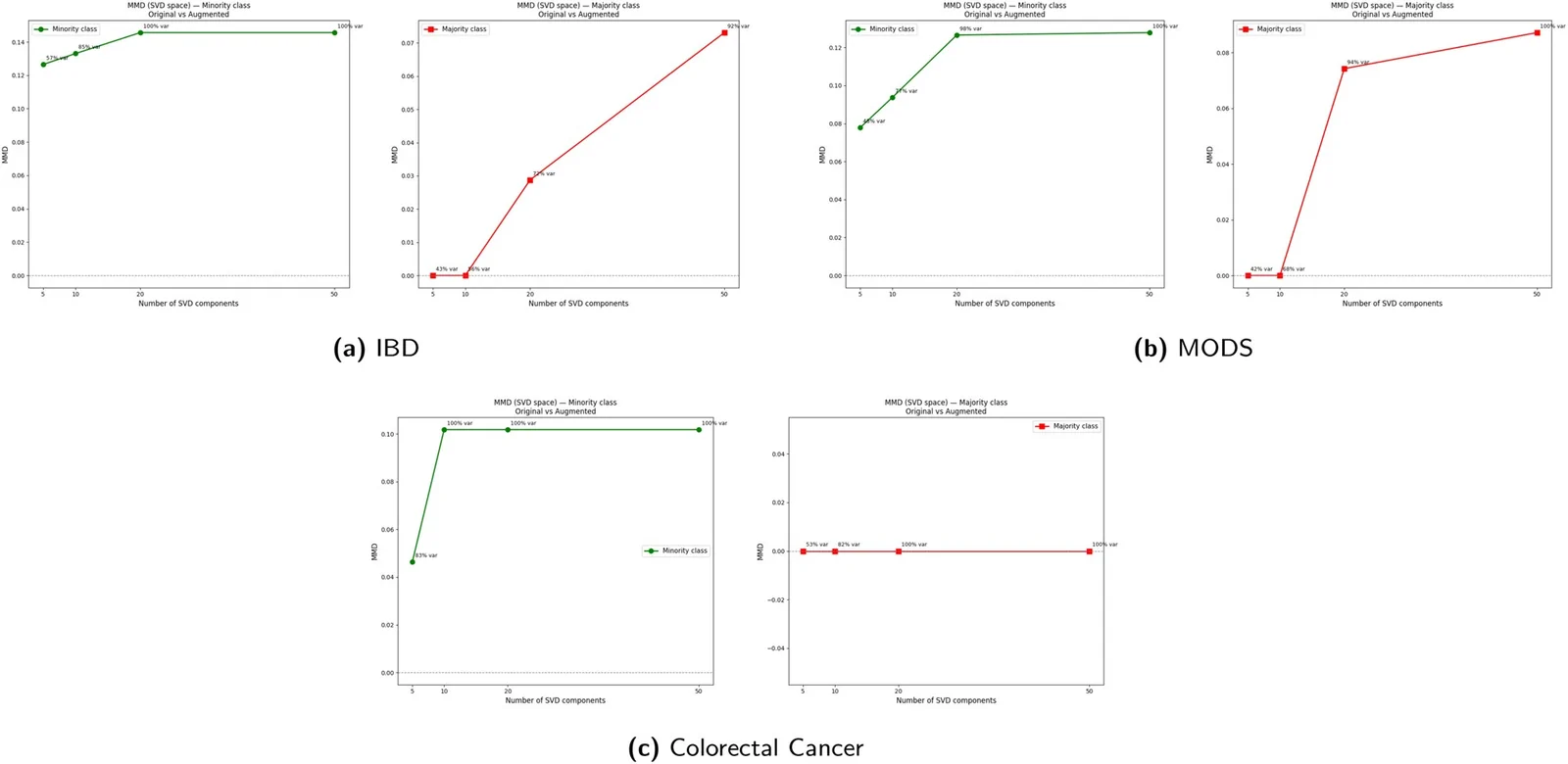
Results of distributional shift incurred due to synthetic sample generation for the three datasets. Figures (a), (b), and (c) refer to IBD, MODS and CC respectively.

The results of MMD-based distributional shift analysis are shown in Fig. 6. They show that TSMmOTE preserves distributions of both minority and majority classes to a high degree across all three datasets, with the magnitude of shift remaining small in absolute terms throughout. For the minority class, MMD increases with the number of SVD components and stabilizes when full variance is captured, settling at a bounded value (0.146 for IBD, 0.128 for MODS, 0.102 for CC). This upper limit indicates a non-escalating shift, which is in congruence with the design intent of three-point interpolation. It shows that the minority-class augmentation introduces deliberate diversity rather than reproducing existing points (a vanishing MMD would instead signal synthetic collapse onto the originals).

The majority class shows a substantially smaller shift in absolute terms across all three datasets. For IBD and MODS, majority-class MMD is indistinguishable from zero while variance capture is below roughly 70%, and only becomes measurable once near-complete variance is retained, peaking at 0.087 and 0.073 respectively, less than two-thirds the magnitude of the corresponding minority-class shift in the same dataset. For CC, majority-class MMD remains near zero at every component count. This pattern is consistent with the SVD-space sensitivity of MMD: at low component counts, the projection is too coarse to resolve small distributional differences, and the metric only becomes informative once the representation is complete. Taken together, the consistently smaller and still low majority-class shift relative to the minority class across all datasets confirms that TSMmOTE’s framework preserves the distributional structure of the synthetic majority class more tightly than the synthetic minority class.

### 3.4 Ablation study

**Figure 7:**
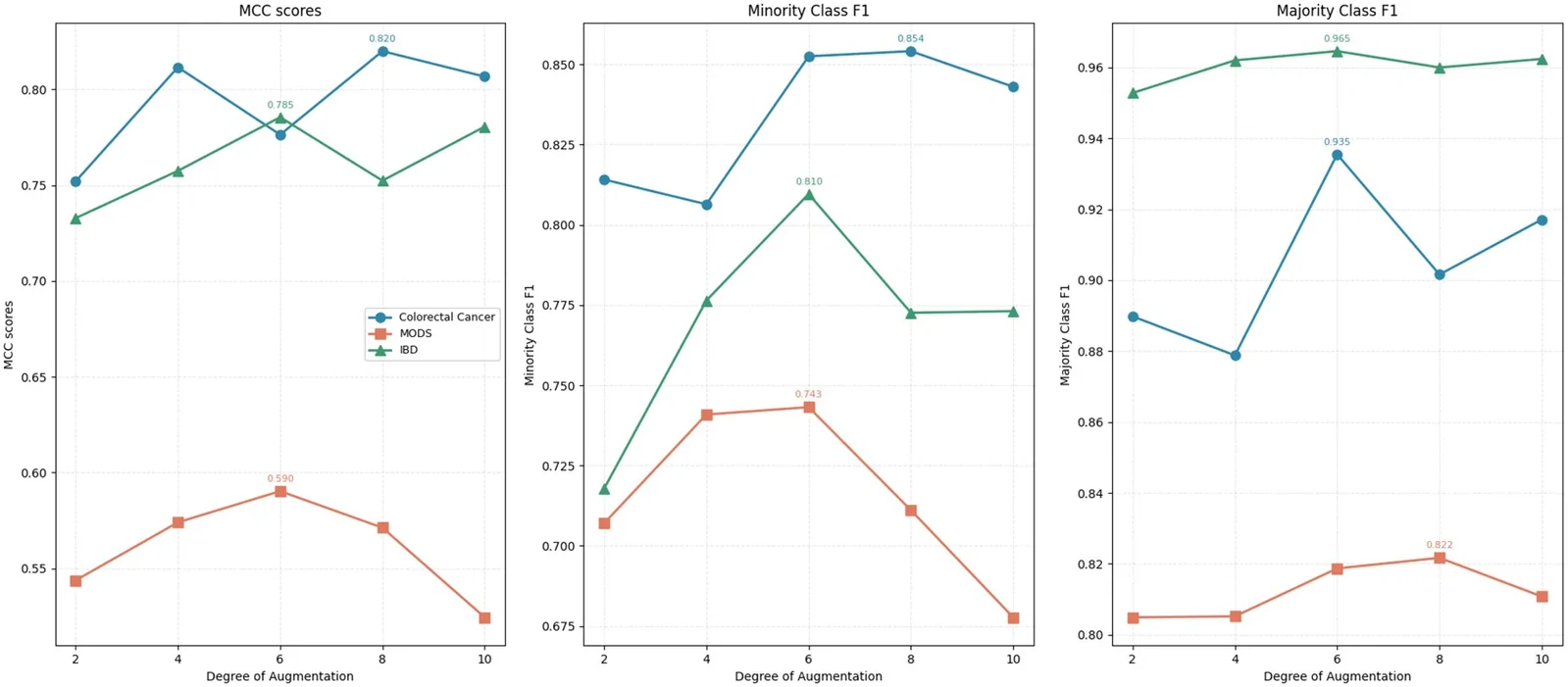
The plots show the performance on the three datasets across various degrees of augmentation.

The first segment of the ablation study explores the relation between degree of augmentation and performance across the datasets. The datasets included in the study shows substantial degree of quantitative and qualitative divergence, and the ablation is conducted to understand the role of augmentation in each. Figure 7 presents the results across all three metrics for CC, MODS, and IBD. Across all three datasets, performance does not improve monotonically with the degree of augmentation. On the contrary, each dataset exhibits a clear peak at an intermediate degree, beyond which performance declines for the chosen range. A degree of 6× emerges as the most consistent optimum across datasets and metrics. IBD peaks at 6× on all three metrics (MCC: 0.785, minority F1: 0.810, majority F1: 0.965), and MODS peaks at 6× for MCC (0.590) and minority F1 (0.743), while its majority F1 peaking marginally later at 8× (0.822). CC presents a slightly divergent outcome, tolerating a somewhat higher degree of augmentation before performance degrades, with MCC (0.820) and minority F1 (0.854) peaking at 8×, while majority F1 peaks at 6× (0.965). The inverted-U pattern shown in the figures suggests a dataset-specific saturation point augmentation beyond which further argumentation introduces redundancy that mildly degrades generalization. For the datasets we have explored, 6× serves as a reasonable default and 8× serves as a viable alternative for augmentation. Notably, majority-class F1 is markedly more stable across augmentation degrees than minority-class F1 or MCC for all three datasets, indicating that majority-class augmentation is more robust to oversaturation, whereas minority-class performance and MCC scores remain more sensitive. These findings motivate us to treat the degree of augmentation as a dataset-specific, tunable hyperparameter rather than a fixed setting in the TSMmOTE framework.

**Figure 8:**
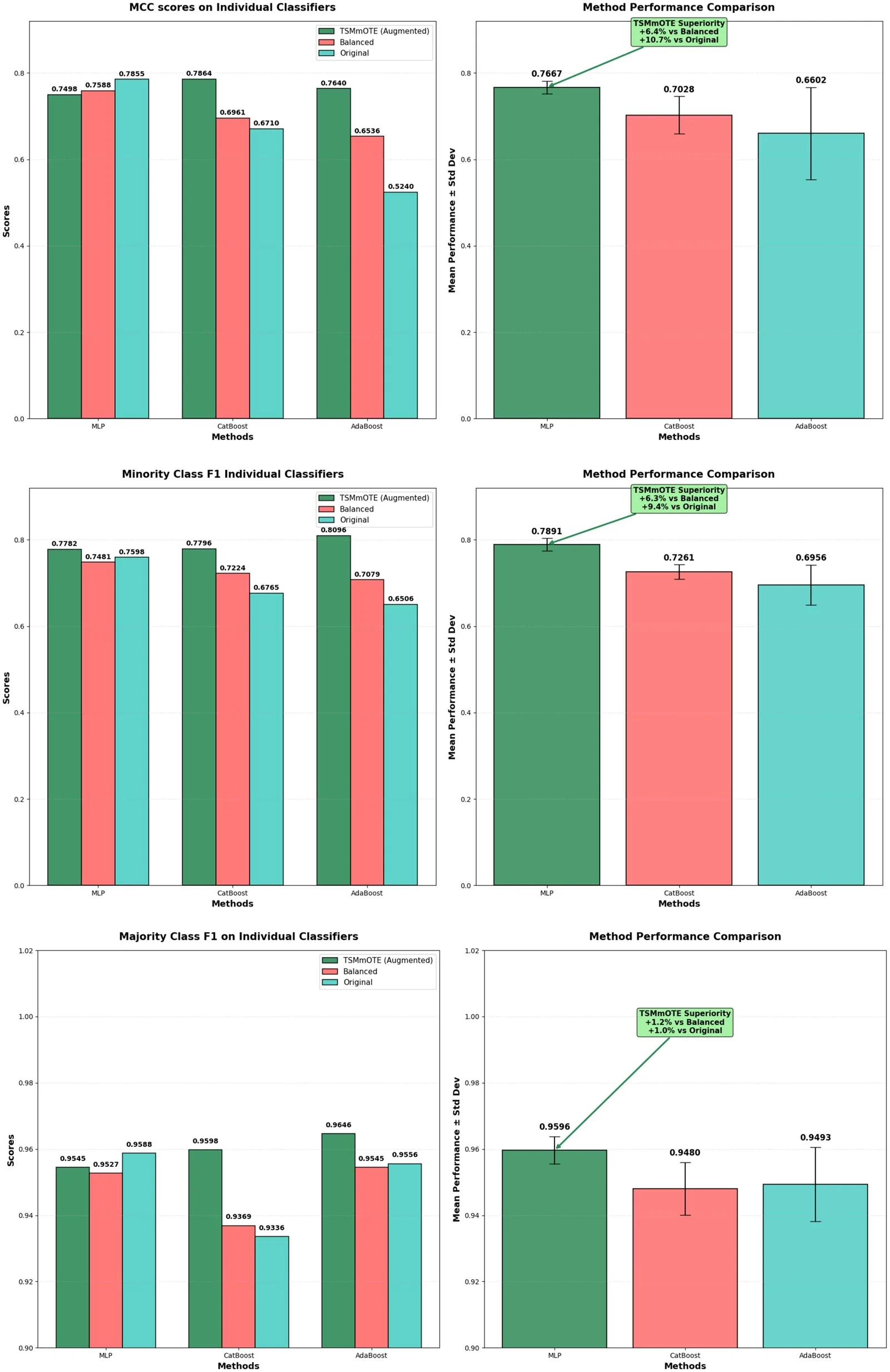
Results of ablation study on *IBD* dataset. The topmost plot shows MCC scores, followed by minority-class $F_{1}$ in the middle plot and majority-class $F_{1}$ at the bottom plot.

**Figure 9:**
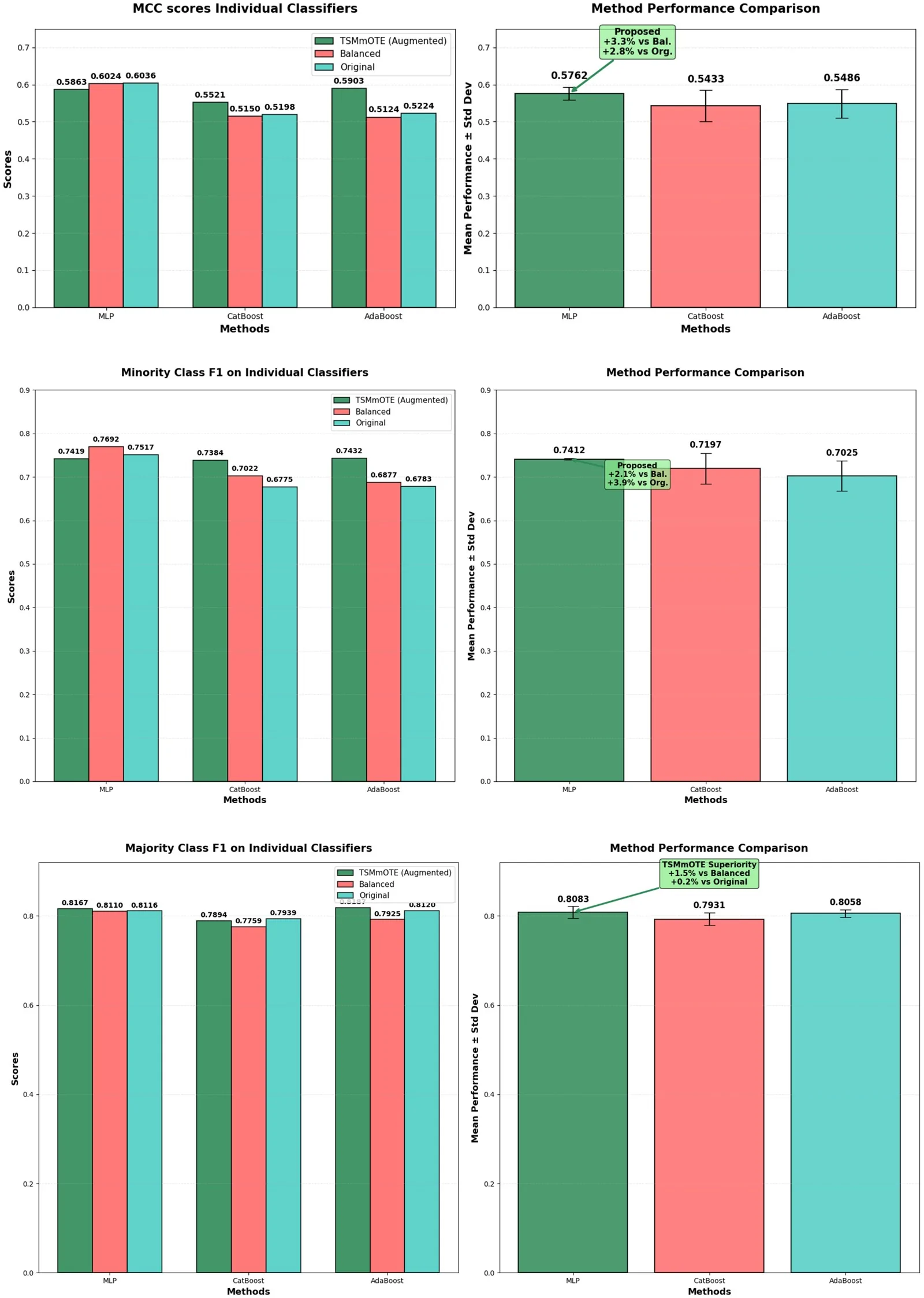
Results of ablation study on *MODS* dataset. The topmost plot shows MCC scores, followed by minority-class $F_{1}$ in the middle plot and majority-class $F_{1}$ at the bottom plot.

**Figure 10:**
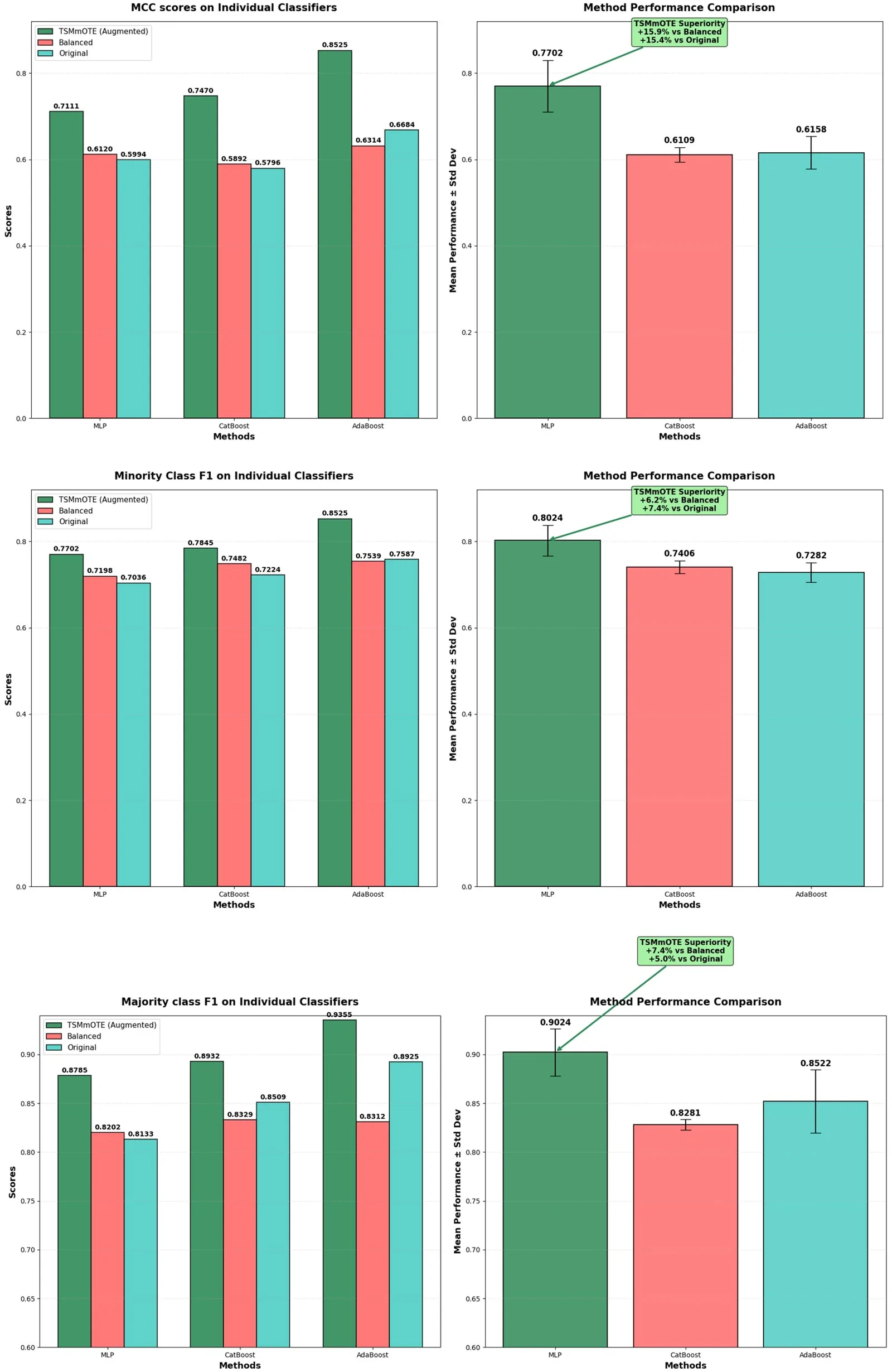
Results of ablation study on *Colorectal Cancer* dataset. The topmost plot shows MCC scores, followed by minority-class $F_{1}$ in the middle plot and majority-class $F_{1}$ at the bottom plot.

To evaluate the contribution of each stage in the TSMmOTE framework, we conduct one more ablation study, and the tabular results are given in the Figs 8–10. TSMmOTE comprises two sequential stages: an intermediate stage that renders a class-balanced dataset (with diverse synthetic samples generated through three-way SMOTE) and a final stage that produces the fully augmented dataset. Accordingly, we train models on three datasets: the original data, the intermediate (balanced) data, and the final (augmented) data. The design isolates the utility of each stage, quantifying the value added by each. Across all three datasets, the models trained on the penultimate TSMmOTE dataset consistently achieve the best cumulative performance when aggregating three classifiers over three diverse evaluation metrics. On the IBD dataset, the final-stage models outperform those trained on the intermediate and original datasets in eight of the nine model–metric combinations; the only exception is the majority-class $F_{1}$ score under the MLP classifier, where the original data performs slightly better. On the MODS dataset, the advantage is even clearer: the final-stage TSMmOTE models achieve the best results across all nine model–metric combinations, thereby establishing the overall utility of the full two-stage oversampling protocol. We observe a scenario similar to IBD in the CC dataset; the performances obtained from the penultimate stage of TSMmOTE outrun the intermediate and original datasets in all but one case. The exception is the majority class $F_{1}$ from the AdaBoost Classifier; the original dataset obtains the best score.

The outcomes validate our core methodological rationale: generating synthetic samples for not only the minority class but the majority class as well. In low-sample size multiomics dataset, exclusive oversampling for the minority class can introduce distortion, which is highly detrimental to the learning of the model. The consistent superiority of the complete two-stage protocol shows that synthesizing samples for the majority class is a critical component for mitigating the distortion and improving generalizable learning of multiomics datasets.

### 4 Discussion

The modus operandi of TSMmOTE is dedicated to addressing a distinct challenge in multi-omics datasets: low sample size. To address the low sample-size problem in multi-omics datasets (a factor that undermines model robustness regardless of class distribution), TSMmOTE implements a novel majority-class oversampling protocol. While this approach of augmenting the majority class may seem counterintuitive, its purpose is to enrich the entire feature representation, providing a more stable and informative data for subsequent classifier training. The results validate this design: the integrated application of minority-class and majority-class oversampling in TSMmOTE yields significant performance gains across overall model performance (MCC scores), majority-class performance (majority-class $F_{1}$), and, crucially, minority-class performance (minority-class $F_{1}$). This outcome substantiates the utility of TSMmOTE in low-sample, imbalanced multi-omics substrates. It further suggests that systematically crafted majority oversampling can enhance, rather than diminish, the learnability of minority class’s footprints as well as majority class’s footprints in high-dimensional spaces.

In the subproblem related to the IBD dataset, TSMmOTE’s superior performance demonstrates its potential as a tool for non-invasive diagnostics via fecal multi-omics. It distinguishes Crohn’s/ulcerative colitis subtypes and controls despite continental variability. High MCC and enhanced $F_{1}$ scores for both classes validate reliable discrimination of Crohn’s disease (CD) from ulcerative colitis (UC) via distinct signatures like CD-enriched fecal calprotectin-linked microbes and UC-depleted short-chain fatty acid producers. This enables early detection in at-risk populations, reduces reliance on biopsies, and enables non-invasive diagnosis. In addition, the cross-continental integration (and subsequent success) demonstrates TSMmOTE’s robustness to dietary, genetic, and environmental factors that alter microbiomes, such as higher Western fat intake, which boosts bile-tolerant pathogens. TSMmOTE’s modus operandi filters noise despite rendering generalizable classifiers for global clinics.

In the MODS-focused subproblem, integrating TSMmOTE with a neural network-based classifier or CatBoost Classifier demonstrates excellence in handling high-dimensional omics data and provides effective early flags. These can be used to reduce ICU burden, ventilator occupancy, and costs in polytrauma; they identify responders to immunomodulators targeting trauma-induced immunosuppression. A substantial increase in minority $F_{1}$ scores ensures that MODS cases are not missed by the tool and paves the way for real-time AI use in AI settings.

The efficiency of TSMmOTE in dealing with CC paves the way for screening via accessible stool proteomics, microbiota, and amino acids, integrated with age, gender, and smoking. This can enable home-based tests to challenge invasive tests like colonoscopy. Fecal multi-omics captures tumor-specific shifts, such as microbial dysbiosis, proteome alterations (mucus proteins), amino acid imbalances, and pre-bowel preparation, sidelining the invasiveness and risks of endoscopy. This can further reduce the cost of diagnosis and remove access barriers.

Notably, the modeling is performed on ultra-low sample size omics profiles. Scaling to a moderately larger cohort can further accentuate TSMmOTE’s utility and the veracity of its predictions. The outcomes across the IBD, MODS, and CC scenarios substantiate TSMmOTE’s role as a non-invasive solution for diagnosing all three health outcomes.

## 5 Conclusion

This research addresses two fundamental problems in multi-omics data: (i) small sample size, and (ii) lack of diversity in the synthetic samples. To this end, we propose a novel scheme, TSMmOTE. Extending the issues addressed in previous research, TSMmOTE proposes a synthetic sample generation strategy for not only the minority class but the majority class as well.

We explore different multi-omics datasets that vary in terms of health conditions and technical parameters, such as imbalance ratios and constituent classes. We explore datasets related to IBD, MODS, and CC. The consistent improvement in performance delivered by TSMmOTE over seven existing methods demonstrates that it is not limited to being a dataset-specific solution; it provides a generalizable, robust framework for enhancing model resilience in data-scarce environments. The outcomes from distribution-fidelity experiment show that majority-class augmentation preserves the original distribution closely. The minority-class augmentation introduces a small, bounded, and stable divergence which is admissible from the aspect of diversity. This consistency across all the datasets (included in the study) reinforces the veracity of TSMmOTE’s synthetic samples to the underlying class-specific data manifold.

The ablation study compares models’ performances trained on the original dataset, the intermediate balanced dataset, and the final augmented dataset from TSMmOTE. The results show that the introduction of synthetic samples for both classes and the diversification of the feature space are critical, sequential contributors to overall performance gains.

The results substantiate the potential of TSMmOTE for the non-invasive detection of an array of diseases using multiomics features. This capability can enable early detection and more accurate diagnoses from limited clinical samples, thereby improving accessibility to advanced diagnostic models and scope for personalized therapies. In the future, we plan to release a Python package for TSMmOTE.

## Data Availability

Code is available in https://github.com/payelu/TSMm.

## Appendix 1. Statistical foundation

In this section, we provide the theoretical underpinnings of TSMmOTE. The foundation addresses three interconnected aspects: the geometric advantage of three-point interpolation, the statistical benefit of increased sample sizes, and the distributional convergence properties of the augmented dataset. We additionally discuss how these classical results are operationalized in a high-dimensional setting through the dimensionality-aware design of TSMmOTE.

### A1.1 Three-point interpolation: local geometric advantage

Standard SMOTE and its variants generate synthetic samples as convex combinations of two original points:

$$H_{2}=\left\{\lambda x_{1}+(1-\lambda )x_{2}:\lambda \in [0,1]\right\}$$

This constrains synthetic samples to a one-dimensional manifold—the line segment connecting $x_{1}$ and $x_{2}$—which has zero Lebesgue measure in any ambient space of dimension greater than one. TSMmOTE instead generates synthetic samples as convex combinations of three original points:

$$H_{3}=\left\{\lambda x_{1}+\mu x_{2}+\nu x_{3}:\lambda ,\mu ,\nu \geq 0,\;\lambda +\mu +\nu =1\right\}$$

$H_{3}$ is a two-dimensional simplex—the interior of the triangle formed by $x_{1}$, $x_{2}$, and $x_{3}$—and satisfies $H_{2}\subset H_{3}$, with the inclusion being strict. The Lebesgue measure of $H_{3}$ is strictly positive in $R^{p}$ for p≥2, while that of $H_{2}$ is zero. This is not merely a quantitative improvement but a qualitative one: three-point interpolation achieves interior coverage of the local feature space, whereas two-point interpolation is confined to a boundary.

We emphasize that this is a local geometric argument. $H_{3}$ is defined with respect to a point and its two nearest neighbors, not with respect to all data points. TSMmOTE does not claim to recover the global convex hull of the dataset—defined as the smallest convex set containing all data points—through three-point interpolation. Rather, it claims to populate the interior of locally representative triangular regions, thereby generating synthetic samples that are both diverse and locally faithful to the data manifold.

### A1.2 Rationale for three-point interpolation

The restriction to three points is a conscious choice grounded in geometric, statistical, and practical considerations, rather than an arbitrary one.

Geometrically, the transition from k=2 to k=3 is the minimal extension that achieves a qualitative improvement in coverage: from a one-dimensional manifold to a two-dimensional simplex with strictly positive measure. Further extensions to k=4 or more points would yield higher-dimensional simplices. Statistically, the choice of k=3 is well-suited to the HDLSS regime that characterizes multi-omics datasets. In high-dimensional spaces with very few samples, the concentration of measure phenomenon causes pairwise distances to concentrate around their mean, making nearest-neighbor relationships increasingly unreliable as the neighborhood size grows (Lualdi and Fasano 2019, Shin and Oh 2024). The three-point scheme uses one pivot point and its two nearest neighbors, keeping the interpolation neighborhood as compact and statistically reliable as possible. Extending to k≥4 would require incorporating neighbors at the periphery of this local neighborhood, where distance relationships are less trustworthy in high dimensions, potentially introducing distributional noise into the synthetic samples rather than faithful local coverage.

Practically, the three-point scheme introduces no additional hyperparameters beyond those already present in standard SMOTE (number of neighbors, number of synthetic points), maintaining the parameter-light character of the framework while delivering improved geometric coverage. A generalization to *k*-point interpolation is a natural direction for future work, particularly as dataset sizes grow and neighborhood structures stabilize; we leave this as an open extension of the current framework.

### A1.3 Variance reduction through augmentation

The Law of Large Numbers (LLN) provides a foundational motivation for synthetic data augmentation. For independent and identically distributed (i.i.d.) random variables $X_{1},X_{2},\ldots ,X_{n}$ with finite expectation $\mu =E[X_{i}]$, the Weak LLN states:

$${\bar{X}}_{n}=\frac{1}{n}\sum_{i=1}^{n}X_{i}\overset{P}{\to }\mu \;\text{as}\;n\to \infty$$

and the Strong LLN gives almost sure convergence:

$$P\left(\underset{n\to \infty }{\text{lim}}{\bar{X}}_{n}=\mu \right)=1$$

Increasing the effective sample size from *n* to n+|S|, where S is the set of synthetic samples, reduces estimation variance. For an estimator ${\hat{\theta }}_{n}$ based on *n* samples:

$$\text{Var}({\hat{\theta }}_{n+|S|})\approx \frac{\text{Var}({\hat{\theta }}_{n})}{1+\frac{\left|S\right|}{n}},\;\text{MSE}({\hat{\theta }}_{n+|S|})\approx \frac{\text{MSE}({\hat{\theta }}_{n})}{1+\frac{\left|S\right|}{n}}$$

This follows from the standard variance-sample size relationship:

$$\text{Var}({\bar{X}}_{m})=\frac{\sigma^{2}}{m}\;\Rightarrow \;\frac{\;\text{Var}({\bar{X}}_{n})}{\text{Var}({\bar{X}}_{n+s})}=\frac{n+s}{n}$$

Note that these results hold under the i.i.d. assumption, which synthetic samples (particularly in very high dimensions) generated by interpolation do not strictly satisfy. The LLN results, therefore, serve as theoretical motivation for augmentation under idealized conditions rather than as dimension-free performance guarantees. The benefits in the HDLSS setting are addressed in Section A1.4 and A1.5 below.

### A1.4 Distributional convergence

Let *F* be the true underlying distribution and ${\hat{F}}_{n}$ the empirical distribution from *n* samples. The Glivenko-Cantelli theorem guarantees uniform convergence of the empirical CDF to the true CDF:

$$\underset{x\in R}{\text{sup}}|{\hat{F}}_{n}(x)-F(x)|\overset{a.s.}{\to }0$$

With synthetic augmentation yielding ${\hat{F}}_{n+s}$ where s=|S|, the rate of convergence improves according to the Dvoretzky-Kiefer-Wolfowitz (DKW) inequality:

$$P\left(\underset{x\in R}{\text{sup}}|{\hat{F}}_{n}(x)-F(x)|>\epsilon \right)\leq 2e^{-2n\epsilon^{2}}$$

$$P\left(\underset{x\in R}{\text{sup}}|{\hat{F}}_{n+s}(x)-F(x)|>\epsilon \right)\leq 2e^{-2(n+s)\epsilon^{2}}$$

Thus, the probability of large deviations from the true distribution decreases exponentially with increased sample size. We acknowledge that the Glivenko-Cantelli theorem and the DKW inequality are classical univariate results. Their direct application to the high-dimensional multi-omics setting requires care, as discussed in the following section.

### A1.5 Dealing with high dimensions

The classical results above motivate augmentation under idealized, low-dimensional conditions. In practice, multi-omics datasets exhibit a high-dimensional, low-sample-size (HDLSS) structure in which p≫n, introducing several challenges that the classical framework fails to address. First, in high dimensions, the concentration of measure phenomenon causes pairwise distances to become increasingly similar, destabilizing nearest-neighbor relationships that interpolation-based methods rely on (Lualdi and Fasano 2019). Second, uniform coverage of a high-dimensional space requires exponentially more samples than a low-dimensional one, meaning that the distributional convergence guaranteed by Glivenko-Cantelli and DKW may require sample sizes that are infeasible in the multi-omics setting. Third, naive interpolation in the ambient high-dimensional feature space risks generating biologically implausible synthetic profiles that distort inter-feature correlations and intrinsic data structures.

TSMmOTE addresses these challenges through two dimensionality-aware mechanisms that are built into its design. The first is SVD-based dimensionality reduction prior to all distance-based computations. As described in Section 2, we project each class independently onto a compact subspace retaining 90% of class-specific variance before performing RkNN analysis and Wasserstein-based subset selection. This ensures that all structural computations—nearest-neighbor identification, distributional alignment assessment, and elite subset selection—are performed in a variance-preserving low-dimensional space where distance relationships are geometrically meaningful. The SVD basis is fitted exclusively on the original class samples, rendering the reduced representation invariant to sample size differences between original and synthetic pools and ensuring that the projection reflects the intrinsic structure of the original data.

The second is Wasserstein-based distributional filtering. Once the synthetic pool is generated, TSMmOTE does not select synthetic points uniformly or at random. Instead, it evaluates each candidate subset against the RkNN lattice of the original class distribution in the reduced space (retaining only those subsets and subsequently those points whose distribution is closest to the representative regions of the original class). This step adds immunity against the generation of distributionally implausible synthetic instances, a failure mode that becomes increasingly severe with dimensionality.

### A1.6 Practical implications

Models trained on the augmented dataset R achieve improved performance because the larger effective sample size $n_{\text{eff}}=n+|S|$ stabilizes gradient estimates and reduces overfitting. For a loss function L(θ), the gradient estimate variance scales as:

$$\text{Var}\left(\nabla_{\theta }L(\theta )\right)\propto \frac{1}{n_{\text{eff}}}$$

This directly impacts optimization stability and generalization. Furthermore, the class-specific augmentation of both majority and minority classes in Stage 2 ensures that the classifier receives enriched intra-class coverage for both classes simultaneously, improving the learnability of class boundaries in the high-dimensional feature space. The empirical evidence supporting these claims is presented in the Results section.
